# Music Listening in Medicine and Healthcare: A Scoping Review

**DOI:** 10.3390/healthcare14091256

**Published:** 2026-05-06

**Authors:** Alfredo Raglio, Virginia Cavallari, Joanna Carvelli, Federica Grossi, Marina Rita Manera

**Affiliations:** Istituti Scientifici Maugeri IRCCS, 27100 Pavia, Italy; cavallarivirginia32@gmail.com (V.C.); joanna.carvelli99@gmail.com (J.C.); federica.grossi@icsmaugeri.it (F.G.); marina.manera@icsmaugeri.it (M.R.M.)

**Keywords:** music listening, music therapy, music medicine, clinical outcomes, music structures and parameters

## Abstract

**Background**: Music listening is increasingly applied in medical and healthcare settings as a non-pharmacological intervention to modulate psychophysical outcomes such as anxiety, pain, stress, mood, and physiological parameters. Despite a rapidly expanding evidence base, receptive music-based interventions remain highly heterogeneous with respect to theoretical rationale, music design, and methodological rigor. **Objective**: The primary aim of this review was to critically examine methodological and conceptual limitations of music listening approaches (based on pre-recorded music listening, without the presence of the music therapist during the listening phase) and to map the range of such interventions across clinical domains. **Methods**: A systematic search of PubMed was independently conducted by two reviewers for randomized controlled trials published between January 2020 and December 2025. Eligible studies investigated psychophysical outcomes of pre-recorded music listening in clinical or medical populations. Studies involving music listening in relational settings, live music, multimodal interventions, or neuromotor rehabilitation were excluded. **Results**: Of 280 records initially identified, 63 studies met the inclusion criteria. Most studies employed conventional familiar music, frequently self-selected by participants. Fewer than half reported explicit musical parameters, and only five studies documented the involvement of a certified music therapist. Substantial heterogeneity was observed in music listening experiences, potentially confounding outcome interpretation. **Conclusions**: Although music listening interventions appear feasible and potentially beneficial across diverse clinical contexts, major methodological and conceptual limitations persist. Greater involvement of music therapy professionals, standardized reporting of musical parameters, clearer theoretical rationales linking musical structure to clinical outcomes, and improved control group design are required to enhance reproducibility, interpretability, and clinical translation.

## 1. Introduction

Music therapy is a non-pharmacological clinical intervention aimed at reducing, stabilizing, or preventing symptoms of mental, physical, cognitive, behavioral, communicative–relational, and social nature, as well as complications associated with those conditions [1]. Its therapeutic effectiveness derives from the intentional and theoretically grounded use of sound- and music-based experiences delivered by a trained music therapist within a clearly defined clinical framework [1]. Music therapy is applied across preventive, rehabilitative, and therapeutic domains. In preventive contexts, musical elements are specifically employed to support interpersonal engagement, emotional expression and regulation, and psychophysical integration [1,2]. Music therapy is conventionally classified into two main modalities: active and receptive (listening-based) techniques. Regardless of modality, effective clinical implementation requires a clearly articulated therapeutic rationale, explicit eligibility criteria, an appropriate treatment setting, operationalized therapeutic goals, methodologically coherent techniques grounded in theoretical models, and validated outcome measures [1]. As an evidence-based discipline, music therapy entails the systematic and intentional application of musical stimuli, either within a therapeutic relationship or through biological and physiological mechanisms.

Active music therapy employs music as a relational medium through which communication, affective modulation, and therapeutic change are co-constructed in real time between therapist and patient. Interventions typically involve improvisational techniques, whereby the therapist contains, mirrors, and modulates emergent musical material to regulate emotional and behavioral states. Delivered according to structured therapeutic protocols, these approaches require the patient’s active participation in music production and are used both preventively and in rehabilitation contexts [3,4]. Despite differences among theoretical models, such as Nordoff–Robbins and Benenzon approaches [5,6], analytic music psychotherapy [7], and cognitive–behavioral music therapy [8], they converge on a shared principle: therapeutic change is mediated by the music-based therapist–patient relationship. In contrast, listening-based approaches encompass two conceptually distinct frameworks that are often insufficiently differentiated in the literature: receptive music therapy and music listening interventions. Receptive music therapy is a relational, therapist-mediated intervention in which the music therapist is actively present during the listening experience and supports the patient in processing emotional, cognitive, and symbolic content elicited by the music. A paradigmatic example is the Bonny Method of Guided Imagery and Music (GIM) [9]. Developed by Helen Bonny, GIM aims to guide clients through expanded states of awareness to access symbolic material, enhance self-understanding, and foster personal growth. Its clinical utility has been documented across a wide range of therapeutic and non-clinical contexts [10]. In this modality, the therapeutic effect emerges from the dynamic interplay between music, therapist, and patient. By contrast, music listening interventions (often referred to as “music medicine” [11]) are non-relational approaches in which individuals listen autonomously to pre-recorded music without the therapist’s co-presence during the listening phase. In these cases, music itself acts as the primary therapeutic agent, modulating physiological, emotional, and cognitive states. However, the role of the music therapist remains crucial, albeit less visible: the therapist is responsible for designing the listening program, selecting musical material based on therapeutic goals and patient characteristics and tastes, and defining the structural parameters of the intervention. A structured example within this framework is therapeutic music listening (e.g., TML) [12], in which playlists are developed by trained music therapists and tailored to individual clinical profiles. In some cases of music listening experience, music is selected directly by the patient (e.g., preferred music) or by healthcare staff, yet even in these scenarios, the therapeutic validity of the intervention depends on how well music selection aligns with clinical objectives and patient’s music identity. The therapeutic value and potential of music listening are supported by a growing body of evidence showing its capacity to modulate physiological and neurochemical activity [13,14], enhance functional connectivity, and promote cognitive and emotional recovery [13,15,16]. Compared to more resource-intensive interventions, music listening is non-invasive and can be tailored to individual preferences, thereby increasing engagement and adherence. Importantly, in several clinical conditions (e.g., in hospital setting) [17] where music listening is more readily implementable than active music-based interventions, as it requires minimal infrastructure and can be delivered even in contexts with constrained resources or reduced patient mobility. Autonomous music listening (without the presence of a music therapist during music listening experience), in addition to being more easily implementable, allows patients to engage with the intervention as needed and promotes greater exposure to the musical stimulus, potentially enhancing its therapeutic effects. These features make it a highly scalable and accessible approach, with promising translational potential for clinical practice [9].

Despite increasing empirical support, a critical issue concerns the limited specification of musical parameters (e.g., tempo, mode, timbre, rhythmic structure) and their mechanistic relationship to targeted outcomes, highlighting the need for more rigorous and replicable/theoretical-based intervention models. The present scoping review focuses specifically on receptive, non-relational music listening interventions involving autonomous exposure to pre-recorded music. The primary objective is to critically examine methodological and conceptual limitations of these approaches and to map the range of such interventions across clinical domains. This scoping review aims not only to provide a comprehensive overview of music listening-based interventions across clinical contexts (including the more recent studies), but also to advance the field by proposing preliminary recommendations to enhance both the scientific quality and the therapeutic potential of music listening approaches. These include the systematic characterization of musical features, the explicit definition of therapeutic mechanisms, and a more clearly articulated role for the music therapist in the construction of listening programs. Strengthening these aspects is essential to improve reproducibility, facilitate comparability across studies, and ultimately support the development of more targeted, theory-driven, and clinically effective music-based interventions.

## 2. Materials and Methods

A systematic search of the PubMed database was conducted independently by two researchers. To focus on the most recent evidence and current reporting standards, the search included articles published between 1 January 2020, and 31 December 2025. Only randomized controlled trials published in English and appearing in peer-reviewed academic journals were eligible for inclusion. The keywords used were: “music listening”, “algorithmic music”, “receptive music therapy” and “playlist”. All medical and clinical domains in which music was applied as the sole intervention were considered, with primary emphasis on pre-recorded music listening interventions delivered without the presence of a music therapist or researcher during the listening experience. Only studies comparing music listening with standard of care interventions were included. Studies based on specific receptive music therapy techniques involving directly the interaction with a music therapist or researcher, live music listening interventions, multimodal interventions combining music listening with other therapeutic components, and studies investigating music listening within neuromotor rehabilitation contexts were excluded from this review.

This Scoping Review was conducted and reported in accordance with the PRISMA guidelines.

**Figure 1:**
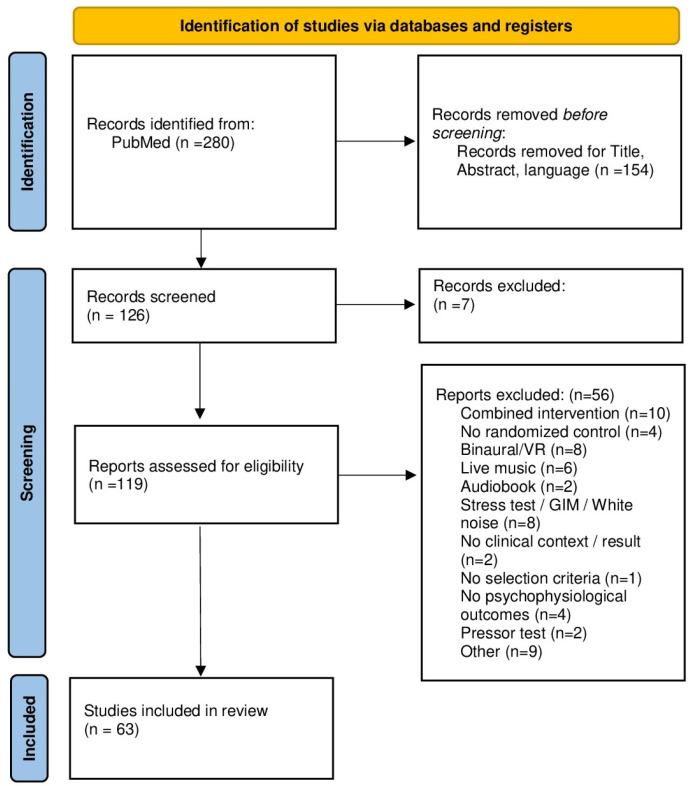
Flow chart of the study.

The Flow Chart of the study is reported in Figure 1.

## 3. Results

The selection process of the studies is reported in Figure 1.

A total of 63 studies were included in this review.

Sixty-one studies investigating music listening interventions were based on conventional music familiar to participants.

Fewer than half of these studies (n = 30) provided explicit methodological details regarding the musical parameters employed within the intervention protocols. Only five studies explicitly reported the involvement of a qualified music therapist in the design, supervision, or delivery of the intervention. Notably, 23 studies adopted approaches in which the musical material was either entirely self-selected by participants or chosen from a predefined set of options. These findings highlight substantial heterogeneity in intervention design and reporting practices across the included studies.

The following section presents four tables (Table 1, Table 2, Table 3 and Table 4) categorized by the method of musical track selection (e.g., self-selected, self-selected from options, experimenter-selected, and other). For a more detailed examination of the results, readers are referred to Table S1 in the Supplementary Materials.

Of the 63 articles included in the review, nine specifically examined musical listening experiences involving self-selected music. In this context, “self-selected” refers to musical tracks that are entirely chosen by the patient, without any external guidance or predefined selection by researchers or clinicians. The following table, Table 1, presents these articles along with a summary of their main findings.

Sixteen studies examined musical listening experiences involving self-selected music from predefined options. In this context, “self-selected from options” refers to musical tracks chosen by patients from a set of selections provided by researchers or clinicians (e.g., large, precompiled playlists), rather than being entirely freely selected. The following table, Table 2, presents these articles and summarizes their main findings.

However, 37 investigated musical listening experiences involved experimenter-selected music. In this context, “experimenter-selected” refers to musical tracks chosen exclusively by the researcher or therapist. This approach ensures a high level of standardization across participants. The following table, Table 3, presents these articles and summarizes their main findings.

In the end, three studies reported musical listening experiences categorized as “other,” in which the process of music selection was not explicitly specified. In this context, “other” refers to studies where it remains unclear whether the musical tracks were selected by the patient, the therapist, or through a predefined set of options. The following table, Table 4, presents these articles and summarizes their main findings.

## 4. Discussion

The investigation of music-based listening interventions in clinical and research contexts is associated with considerable methodological challenges. Music represents a multidimensional and culturally embedded phenomenon, the perception and effects of which vary substantially across individuals. This intrinsic variability complicates the identification of consistent therapeutic mechanisms and limits the generalizability of findings. Although clinical objectives are often clearly defined, the musical material used to achieve these objectives is frequently described in vague, incomplete, or insufficiently operationalized terms. Within this framework, the limited involvement of certified music therapists constitutes a major methodological limitation. Music therapists are specifically trained to analyze, select, and systematically describe musically relevant properties in relation to clinical goals. The present review revealed a widespread lack of professional oversight not only during intervention delivery but also during critical phases of playlist construction and design. Such omissions weaken scientific rigor by detaching interventions from established theoretical models and evidence-based clinical practice.

Cultural background further shapes the subjective perception of music, influencing emotional engagement, meaning attribution, and therapeutic responsiveness. While the use of familiar or self-selected music is frequently supported in the literature, often justified by neuroscientific evidence indicating enhanced emotional salience [81,82,83], preference-based selection alone does not constitute a sufficient therapeutic rationale [9]. This limitation is particularly relevant when clinical aims require the precise modulation of physiological, emotional, or cognitive states.

A recurrent limitation across the reviewed studies concerns the imprecise reporting of musical parameters. Many investigations rely on broad descriptors such as ‘slow tempo’, ‘relaxing’, or ‘calming’, without specifying structural characteristics including tempo range, rhythmic complexity, harmonic density, timbral qualities, spectral features, or dynamic contour. This lack of detail undermines reproducibility, limits interpretability, and prevents meaningful cross-study comparisons, thereby impeding the identification of stable associations between music structure and clinical outcomes.

Progress in this field requires the development of coherent therapeutic rationales that explicitly link defined music parameters to targeted clinical objectives. Such advancement entails moving beyond descriptive or preference-based frameworks toward hypothesis-driven models that address the functional relevance of musical structure. These hypotheses must be empirically tested through rigorously designed and adequately powered clinical trials.

Possible methodological approaches include therapeutic music listening (TML) and algorithmic music protocols [9], which aim to integrate individualization with systematic control of musical features. By combining predefined parameters with individualized listening profiles, these approaches represent a shift toward greater methodological rigor while maintaining sensitivity to interindividual variability. In this context, the provision of specialized training for music therapists in the use of algorithmic music and emerging musical genres, particularly those generated through artificial intelligence, would be of substantial value [84].

Artificial intelligence offers significant potential for the generation of original musical material characterized by precisely controlled parameters and diverse acoustic features tailored to specific therapeutic objectives. Unlike conventional music selections, algorithmically generated music can be designed with a high degree of precision in terms of tempo, timbre, harmony, and structural complexity, thereby enabling standardized and reproducible auditory stimulation [85]. Only one identified study [18] has employed algorithmic music exclusively for therapeutic purposes, distinguishing it from traditional music-based interventions that are often influenced by cultural background, autobiographical associations, and prior listening habits. This relative independence from personal and cultural conditioning represents a notable methodological advantage and warrants further investigation through large-scale, rigorously designed studies. An additional methodological issue concerns the definition and implementation of control conditions. Although control groups are typically described as receiving standard care, substantial heterogeneity exists with respect to environments to which participants are exposed. In several studies, control participants received standard therapy without any modification, whereas in others they were provided with headphones, with inconsistent reporting regarding the use of active noise cancelation (e.g., ANC).

This variability raises important concerns regarding modifications of the auditory environment and consequently influence on perceptual, cognitive, or emotional states. In fact, auditory input plays a critical role in shaping internal experience and interaction with the external environment; therefore, any alteration in sensory input has the potential to indirectly affect therapeutic outcomes. The use of ANC headphones may further amplify this issue by substantially reducing ambient environmental noise. As this point, music listening condition with ANC could be advisable to facilitate patients’ focused attention on intervention.

We acknowledge as limitations of the present review the restricted time frame (2020–2025) adopted for study inclusion and the use of PubMed as the sole database. The decision to focus on recent years was intended to capture the most up-to-date evidence and methodological advancements in the field, particularly in a rapidly evolving area of research. However, this choice may have led to the exclusion of earlier relevant studies. Similarly, the use of a single database ensured a consistent and manageable search strategy but may have limited the comprehensiveness of the literature retrieval. These aspects should be considered when interpreting the findings of the review.

## 5. Conclusions

In conclusion, this review highlights the need for greater methodological rigor and transparency in studies investigating music listening interventions. Attention should be devoted to the characterization of the listening content, including a clear rationale underlying the selection of musical material, as well as a detailed description of the musical parameters and structural features that define the listening programs in relation to specific therapeutic objectives. Equally important is the specification of listening modalities, such as timing, setting, and the technological supports employed, which should be tailored to both the clinical context and the intended outcomes. From a methodological perspective, careful consideration should also be given to the design of control conditions. Future research would benefit from a greater number of comparative studies examining different types of listening approaches (e.g., self-selected versus experimenter-selected music, algorithmically generated music versus curated playlists, etc.), while maintaining a rigorous experimental framework. In addition, a precise and standardized definition of the music-based intervention—here, music listening—is essential, in line with current guidelines for music-based therapeutic interventions [86]. Moreover, the role of the music therapist remains fundamental, particularly in the design, personalization, and monitoring of the intervention, as well as in ensuring its clinical appropriateness and effectiveness.

Finally, innovative technological approaches, including algorithmic music generation and artificial intelligence-based composition, represent valuable tools for producing precisely controlled yet adaptable musical stimuli. When integrated within clinically grounded theoretical frameworks and supported by interdisciplinary collaboration, these approaches hold significant promise for advancing hypothesis-driven research and the development of robust, evidence-based clinical applications.

## Figures and Tables

**Table 1:** Studies reporting the use of self-selected music.

Articles	Clinical Conditions/Context	Sample Size (Experimental Group/Control Group)	Aim	Type & Duration	Music Parameters (Not Specified, Values Reported, Values Not Reported)	Music Therapist	Main Significant Results
Raglio A et al., 2023 [18]	Fibromyalgia	24 (12/12)	Evaluate therapeutic music listening	Conventional & generated; <5 weeks	Values not reported (music parameters maintained a high level of stability in intensity, variations, duration of notes and range of intervals, creating a low level of stimulation)	Yes	Influence on mental well-being
Kakde A et al., 2023 [19]	Cesarean delivery	110 (53/55)	Anxiety and catastrophizing	Conventional; <5 weeks	Not specified	Not specified	Significant (e.g., Sig.) decrease in postop VAS-A scores, in PCS total scores, in rumination, magnification, helplessness
Saccone G et al., 2024 [20]	Labor	30 (15/15)	Pain and anxiety	Conventional; <5 weeks	Not specified	Not specified	Sig. difference in pain and anxiety
Baltacı N et al., 2024 [21]	Pregnancy	126 (42/41/43)	Anxiety and stress	Conventional; <5 weeks	Values reported (slow rhythm, between 60 and 70 bpm when measured by a metronome); values not reported (soft melody)	No	Sig. decrease in post-test anxiety and stress levels
Karapicak E et al., 2023 [22]	Dental anxiety	70 (35/35)	Dental anxiety	Conventional; <5 weeks	Not specified	Not specified	Sig. decrease in Diastolic Bood Pressure (e.g., DBP) and MDAS scores
Laframboise-Otto JM et al., 2021 [23]	Arthroplasty	97 (24/26)	Pain and analgesic use	Conventional; <5 weeks	Not specified	No	Sig. decrease in pain
Kırdemir P et al., 2025 [24]	Pregnant women	102 (51/51)	Perioperative anxiety	Conventional; <5 weeks	Not specified	Not specified	Sig. change in STAI-SA levels and increase in pain and VAS levels
Lund HN et al., 2023 [25]	Depression-related insomnia	112 (56/56)	Insomnia and quality of life	Conventional; <5 weeks	Not specified	No	Sig. decrease in global scores, insomnia symptoms and improvement in sleep quality and wellbeing
Charron M et al., 2025 [26]	Emergency wound closure	170 (86/84)	Pain and anxiety	Conventional; <5 weeks	Values reported (maximum volume of 60 dB)	Not specified	Sig. decrease in values of respiratory rate (e.g., RR) and anxiety score, improvement in satisfaction

**Table 2:** Studies reporting the use of self-selected music from options.

Articles	Clinical Conditions/Context	Sample Size (Experimental Group/Control Group)	Aim	Type & Duration	Music Parameters (Not Specified, Values Reported, Values Not Reported)	Music Therapist	Main Significant Results
Hoegholt NF et al., 2024 [27]	Pregnancy-related insomnia	106 (31/40)	Evaluate music listening plus sleep hygiene	Conventional; <5 weeks	Values not reported (slow tempo, a stable dynamic structure, and a simple structure in melody, harmony, and rhythm)	Not specified	Sig. difference in adherence to sleep hygiene
Lee AL et al., 2024 [28]	Chronic Obstructive Pulmonary Disease (e.g., COPD) rehabilitation	58 (28/30)	Assess music during pulmonary rehabilitation	Conventional; >5 weeks	Values reported (tempo of 90 beats/minute)	Yes	No Sig. results were found in the study
Wakana K et al., 2022 [29]	Dental surgery	60 (30/30)	Preoperative anxiety	Conventional; <5 weeks	Not specified	No	No Sig. results were found in the study
Yang HF et al., 2024 [30]	Radiotherapy	100 (50/50)	Anxiety during radiotherapy	Conventional; <5 weeks	Not specified	Not specified	Sig. in post-test BAI-C score
Harper FWK et al., 2023 [31]	Chemotherapy	750 (387/352)	Mood and distress	Conventional; >5 weeks	Not specified	No	Sig. improvement in positive mood and distress
Aksu Ç, 2023 [32]	Endoscopy	104 (40/40)	Anxiety, pain, comfort	Conventional; <5 weeks	Values not reported (slow rhythm and relaxing characteristics); values reported (no words)	No	Sig. decrease in STAI-S and VAS-Pain scores
Azi LMTA et al., 2021 [33]	Orthopedic surgery	112 (56/56)	Anxiety and sedation	Conventional; <5 weeks	Values reported (instrumental, mostly classical with less than 72 beats per minute, volume no more than 60 dB); values not reported (bass tone and soft melody)	Not specified	Sig. reduction in anxiety in Music Group (e.g., MG) patients after surgery
Wang J et al., 2024 [34]	General anesthesia	166 (84/82)	Hemodynamics and anxiety	Conventional; <5 weeks	Not specified	Not specified	Sig. improvement of STAI in Music Intervention Group (e.g., MIG)
Sun DJ et al., 2022 [35]	Colonoscopy	216 (112/104)	Satisfaction, anxiety, pain	Conventional; <5 weeks	Not specified	No	Sig. decrease in anxiety and improvements in patient satisfaction and comfort during colonoscopy
Jacquier S et al., 2022 [36]	Intensive care unit catheterization	75 (38/37)	Anxiety during catheter insertion	Conventional; <5 weeks	Not specified	No	No Sig. results were found in the study
Pampel J et al., 2024 [37]	Peripheral vascular interventions	204 (101/103)	Procedure-related anxiety	Conventional; <5 weeks	Values reported (slow tempo with 60 to 80 bpm, instrumental); values not reported (regular rhythm, low pitch)	No	Sig. reduction in Non Stress Test (e.g., NST)
Başkurt H et al., 2024 [38]	Pregnancy	139 (67/63)	NST & anxiety and stress	Conventional; <5 weeks	Not specified	Not specified	Sig. difference in acceleration number, in rate of reactive NST levels Intervention Group (e.g., IG); in STAI scale
Öztürk FU et al., 2023 [39]	Breast biopsy	93	Anxiety, pain, satisfaction	Conventional; <5 weeks	Not specified	Not specified	Sig. decrease in anxiety, pain and satisfaction
Chen YB et al., 2021 [40]	Pelvic reconstructive surgery	70 (35/35)	Preoperative anxiety	Conventional; <5 weeks	Value reported (instrumental)	No	Sig. improvement in state anxiety and satisfaction
Agus Y et al., 2025 [41]	Thyroid surgery	76 (38/38)	Fear and anxiety	Conventional; <5 weeks	Not specified	No	Sig. decrease in ASSQ post-test scores
Fleckenstein FN et al., 2025 [42]	CT-guided procedures	209 (107/102)	Anxiety and pain	Conventional; <5 weeks	Values reported (volume ranging from 50 to 60 dB)	No	Sig. decrease in anxiety

**Table 3:** Studies reporting the use of experimenter-selected music.

Articles	Clinical Conditions/Context	Sample Size (Experimental Group/Control Group)	Aim	Type & Duration	Music Parameters (Not Specified, Values Reported, Values Not Reported)	Music Therapist	Main Significant Results
Raglio A et al., 2023 [18]	Fibromyalgia	24 (12/12)	Evaluate therapeutic music listening	Conventional & generated; <5 weeks	Values not reported (music parameters maintained a high level of stability in intensity, variations, duration of notes, range of intervals, creating a low level of stimulation)	Yes	Influence on mental well-being
Petrovsky DV et al., 2023 [43]	Dementia	37 (17/16)	Feasibility and sleep outcomes in older adults with dementia and their caregivers	Conventional; <5 weeks	Values reported (at least 30 min in length, 36 between 60 and 80 beats per minute (bpm)); values not reported (slow stable rhythm, low-frequency tones, and absence of lyrics or strong percussion)	Yes	Improvement sleep outcomes
Mir IA et al., 2021 [44]	Pre-hypertensive young adults	30 (15/15)	Effect on Blood Pressure (BP) and Heart Rate (HR)	Conventional; <5 weeks	Not specified	Not specified	Sig. reduction in Systolic Blood Pressure (e.g., SBP) and HR
Calamassi D et al., 2022 [45]	Emergency nurses	83 (28/28/27)	Effect on anxiety/stress	Conventional (432/440 Hz); <5 weeks	Values reported (432 Hz, 440 Hz)	No	Decrease in STAI X1; reduction in respiratory rate and SBP
Lázaro-García A et al., 2024 [46]	Stem cell transplantation and acute myeloid leukemia	71 (4; 12; 19; 5/2; 5; 20; 4)	Symptom burden and quality of life	Conventional; >5 weeks	Values not reported (rhythm, melody, harmony); values reported in table (bpm, tonality)	No	Sig. difference in symptoms burden and in proportion of satisfactory sessions
Coşkun Ç et al., 2025 [47]	Prostate biopsy	149 (78/71)	Pain and anxiety	Conventional; <5 weeks	Not specified	No	Sig. difference in VAS score and in median s-STAI score
Hillebrand MC et al., 2023 [48]	Dementia	118 (61/57)	Behavioral and psychological symptoms of dementia (e.g., BPSD) modulation	Conventional; >5 weeks	Values not reported (tonality, tempo)	No	Sig. decrease in BPSD
Hakim A et al., 2025 [49]	Hospitalized children	52 (26/26)	Anxiety reduction	Conventional; <5 weeks	Values reported (non-verbal music)	No	Sig. reduction in anxiety and vital signs
Durgun H et al., 2021 [50]	Cystoscopy	72 (36/36)	Pain and anxiety	Conventional; <5 weeks	Values reported (all instrumental without any words); values not reported (a strong beat or fluctuating rhythms)	No	Sig. decrease in pain scores
Baltacı N et al., 2024 [21]	Pregnancy	126 (42/41/43)	Anxiety and stress	Conventional; <5 weeks	Values reported (slow rhythm, between 60 and 70 bpm when measured by a metronome); values not reported (soft melody)	No	Sig. decrease in post-test anxiety and stress levels
Schaal NK et al., 2021 [51]	Catheter placement	107 (44/40)	Anxiety course	Conventional; <5 weeks	Not specified	No	Sig. reduction in SBP and HR
Miladi S et al., 2024 [52]	Rheumatic diseases	70 (40/30)	Mood outcomes	Conventional; <5 weeks	Not specified	Not specified	Sig. reduction in HR and STAI
Brix LD et al., 2022 [53]	Colonoscopy	337 (169/168)	Effect of MusiCure	Conventional; <5 weeks	Values reported (instrumental acoustic music with integrated sounds of nature)	No	No Sig. results were found in the study
Bürlukkara S et al., 2024 [54]	Intrarenal surgery	286 (144/142)	Pain and anxiety	Conventional; <5 weeks	Values reported (a maximum sound level of 85 dB, instrumental)	Not specified	Sig. decrease in HR, VAS score and STAI score
Kaur H et al., 2022 [55]	Orthopedic surgery	70 (35/35)	Anxiety and hemodynamics	Conventional; <5 weeks	Not specified	Not specified	Sig. decrease in HR; and RR
Ferraz MCL et al., 2021 [56]	Tibial fractures	70 (35/35)	Procedural pain	Conventional; <5 weeks	Not specified	No	Sig. decrease in pain scores
Baykan S et al., 2025 [57]	Fistula needle insertion	56 (28/28)	Pain and distress	Conventional; <5 weeks	Values reported (between 25 and 50 db)	No	Sig. reduction in severity of pain and distress level
Ugurlu M et al., 2024 [58]	Menopausal symptoms	70 (35/35)	Effects on menopause symptoms	Conventional; >5 weeks	Not specified	No	Sig. difference in post-test BDI and PSQI scores and in post-test MRS total, somatic, psychological symptoms scores
Tola YO et al., 2025 [59]	Mastectomy	40 (18/18)	Pain and anxiety	Conventional; <5 weeks	Not specified	No	Sig. difference in pain intensity, anxiety, level of satisfaction in SBP and DBP
Inoue M et al., 2024 [60]	Dementia	261 (148/113)	Mood and behavior	Conventional; <5 weeks	Not specified	No	Sig. increase in total PHQ-9 scores
Baltacı N et al., 2023 [61]	Pregnancy (distress)	192 (50/50/50)	Distress and attachment	Conventional; <5 weeks	Values reported (in the range of 60–70 bpm)	No	Sig. decrease in prenatal distress and antenatal attachment levels
Oyur Celik G et al., 2022 [62]	Coronary angiography	62 (31/31)	Pain and anxiety	Conventional; <5 weeks	Values reported (non-verbal and instrumental music at 60 (Adagio) and 100 (Andante))	No	Sig. difference in anxiety, pain, DBP and Band pulse wave velocity in vital signs
Barcos-Munoz F et al., 2025 [63]	Preterm infants	54 (28/26)	HR variability modulation	Conventional; >5 weeks	Values reported (B major, from 30 dBA for the background to 65 dBA for the peak with bells, average pitch of 319.2 Hz (range 320–360 Hz); 10 dB difference between the ambient sound level and the music level); values not reported (moderato tempo and constant rhythm)	No	Sig. difference in SD1 and SD2 variables
Hillebrand MC et al., 2025 [64]	Dementia	130 (61/57)	Goal attainment	Conventional; >5 weeks	Not specified	No	Sig. increase in goal attainment
Sökmen Y et al., 2024 [65]	Pregnancy	112 (56/56)	NST & satisfaction	Conventional; <5 weeks	Not specified	Not specified	Sig. increase in basal fetal HR and count of fetal movements
Esteban Pellicer LÁ et al., 2023 [66]	Dental implant surgery	33 (8/10/8)	Anxiety and pain	Conventional; <5 weeks	Value reported (volume maximum of 60 dB)	Not specified	Sig. difference in degree of anxiety
Cavnar Helvaci B et al., 2024 [67]	Thyroid fine needle aspiration biopsy (e.g., FNAB)	562 (265/285)	Pain during FNAB	Conventional; <5 weeks	Value reported (nonlyrical, tempo of 60 to 80 beats per minute and a volume of around 60 dB); value not reported (flowing melody, low tones, minimal percussion)	No	Significant decrease in pain scores
Çuhacı AB et al., 2024 [68]	Premature retinopathy (e.g., ROP) examination	29 (15/14)	Pain and comfort	Conventional; <5 weeks	Value reported (volume of 45–60 dB)	Not specified	Sig. decrease in PIPP-R scores and in post-procedure PICS values
Esfahanian F et al., 2022 [69]	Delirium prevention	200 (100/100)	Delirium prevention	Conventional; <5 weeks	Value reported (moderate noise level of 10%, low tempo (60–80 beats/min), and frequency of 440 to 470 kHz)	No	Sig. effect of music in preventing delirium
Toker E et al., 2021 [70]	Postpartum	128 (42/42/42)	Pain and anxiety	Conventional; <5 weeks	Not specified	No	Sig. reduction in anxiety scores and pain levels
Aker N et al., 2024 [71]	Urological surgery	80 (40/40)	Preoperative anxiety	Conventional; <5 weeks	Value reported (instrumental)	No	Sig. difference in STAI-S
Gürkan O et al., 2024 [72]	Thyroid FNAB	94 (47/47)	Anxiety and pain	Conventional (432 Hz); <5 weeks	Values reported (432 Hz)	No	Sig. difference in state anxiety scale
Huang YL et al., 2021 [73]	Congenital heart disease	90 (45/45)	Anxiety and cooperation	Conventional; <5 weeks	Values not reported (rhythm was smooth, the volume was soft)	Not specified	Sig. decrease in Mean Arterial Pressure (e.g., MAP) HR, YPAS-SF scores and in ICC score
Zhang Y et al., 2025 [74]	Post-anesthesia care	194 (97/97)	Recovery outcomes	Conventional; <5 weeks	Not specified	No	Sig. reduction in STAI-S scores
Torlak MS et al., 2025 [75]	Chronic neck pain	40 (20/20)	Pain, anxiety and quality of life	Conventional; <5 weeks	Values reported (70 dB)	Not specified	Sig. difference in the Beck Anxiety Inventory, visual analog scale, Neck Disability Index, and SF-36 physical scores and VAS
Khan BA et al., 2025 [76]	Delirium in Intensive care unit (e.g., ICU)	159 (79/80)	Delirium duration and severity, pain or anxiety	Conventional; <5 weeks	Values reported (80–60 bmp, no lyrics or spoken words)	Yes	No Sig. results were found in the study
Doğan P et al., 2025 [77]	Premature infants	63 (20/21/22)	Pain during heel blood collection	Conventional; <5 weeks	Values reported (50 dB)	Yes	Sig. difference in pain level

**Table 4:** Studies in which music intervention is not specified.

Articles	Clinical Conditions/Context	Sample Size (Experimental Group/Control Group)	Aim	Type & Duration	Music Parameters (Not Specified, Values Reported, Values Not Reported)	Music Therapist	Main Significant Results
Pradit L et al., 2022 [78]	Cervical biopsy pain	240 (80/80/80)	Efficacy in pain reduction	Not specified; <5 weeks	Not specified	No	No Sig. results were found in the study
Hillebrand MC et al., 2024 [79]	Dementia	32 (19/13)	Individualized music effects	Conventional; >5 weeks	Not specified	Not specified	No Sig. results were found in the study
Ezepue CO et al., 2023 [80]	Cataract surgery	98 (49/49)	Preoperative anxiety	Not specified; <5 weeks	Not specified	Not specified	Sig. decrease in anxiety scores

## Data Availability

No new data were created or analyzed in this study.

## Supplementary Materials

The following supporting information can be downloaded at: https://www.mdpi.com/article/10.3390/healthcare14091256/s1, Table S1. Detailed examination of the selected studies’ results.

## Abbreviations

The following abbreviations are used in this manuscript:

NMT	Neurologic Music Therapy
GIM	Guided Imagery and Music
TML	Therapeutic Music Listening
Sig.	Significant
DBP	Diastolic Blood Pressure
RR	Respiratory Rate
COPD	Chronic Obstructive Pulmonary Disease
MG	Music Group
MIG	Music Intervention Group
NST	Non Stress Test
IG	Intervention Group
BP	Blood Pressure
HR	Heart Rate
SBP	Systolic Blood Pressure
BPSD	Behavioral and Psychological Symptoms of Dementia
FNAB	Thyroid Fine Needle Aspiration Biopsy
ROP	Premature Retinopathy
MAP	Mean Arterial Pressure
ICU	Intensive Care Unit
ANC	Active Noise Cancellation
ML	Music Listening (Supplementary Material)
MLG	Music Listening Group (Supplementary Material)
Vs	Versus (Supplementary Material)
f/u	follow up (Supplementary Material)
CG	Control Group (Supplementary Material)
IML	Individualized Music Listening (Supplementary Material)
LG	Listening Group (Supplementary Material)
MT	Music Therapy (Supplementary Material)
