# Supplementary material for: Music Listening in Medicine and Healthcare: A Scoping Review
Source: Healthcare (Basel). 2026 May 6;14(9):1256. doi: 10.3390/healthcare14091256 (PMC13163375; doi:10.3390/healthcare14091256)
Supplement: Supplementary file 1 [file healthcare-14-01256-s001.zip › healthcare-4185302-supplementary.pdf]

| Articles | Main Significant Results |
| --- | --- |
| Raglio A et al., 2023 [18] | Influence on mental well-being by Music Listening (e.g. ML) in merged Music Listening Group (e.g. MLG) Versus (e.g. vs) no Music Group (e.g. MG), (end of treatment p=.004; follow up (e.g. f/u, p=.034) and in MG undergone preferred playlist listening (p=.013) vs no MG; Improvement mental well-being in merged MG interventions vs no MG (MG p=.004; f/u p=.034) |
| Kakde A et al., 2023 [19] | Sig. decrease in postop VAS-A scores in MG (p<.001); Sig. in PCS total scores in MG (p=.003), rumination (p=.035), magnification (p=.006), helplessness (p=.001) sub-scores |
| Saccone G et al., 2024 [20] | Sig. difference in pain level during induction procedure in MG and Control Group (e.g. CG), (p<.01); Sig. of anxiety during induction procedure in MG (p<.01) |
| Baltacı N et al., 2024 [21] | Sig. decrease of post-test anxiety and stress levels in IG vs CG (p<.01); Sig. decrease of post-test anxiety in MG vs MLG (p<.01) |
| Karapicak E et al., 2023 [22] | Sig. decrease in DBP (p=.042) and the MDAS scores of experimental group (p=.001) than the CG at the end of the treatment |
| Laframboise- Otto JM et al., 2021 [23] | Sig. decrease in pain intensity and pain distress (p<.05) |
| Kırdemir P et al., 2025 [24] | Sig. change in STAI-SA levels over time in MG (p<.001), decrease in STAI-SA levels in T0 and T2 vs T1 (respectively p=.006 and p<.001); Sig. difference in STAI-SA levels with time (p<.001);decrease in STAI-SA levels in T2 than in T1 (p<.001) in isolated from ambient noise group (e.g. Group S); Sig. difference in STAI-SA levels with time (p=.009),decrease in STAI-SA levels in T2 vs T1 (p=.011) CG; Sig. similarity in changes in T1 according to T0,T2 according to T0,T2 according to T1 between the groups (respectively p=.979,p=.057,p=.402); Sig. increase in pain levels in MG postop 12th-hours vs the postop 1st-hour (p < 0.001); In Group S, Sig. change in VAS levels over time (p<.001),vs postop 1st hour; postop 8th and postop 12th-hour VAS levels were higher (respectively p=.003 and p<.001); Sig. change in VAS levels over time in CG (p=.003),higher VAS levels in postop 8th-hour than the postop 1st-hour (p=.008) |
| Lund HN et al., 2023 [25] | Sig. decrease in global scores from baseline to 4 weeks in MIG (p<.001); Sig. decrease of insomnia symptoms in MIG at 4 weeks (p<.001); Sig. difference of wellbeing between groups (p=.004) at 4 |

| | |
|---|---|
| | weeks, with no Sig. decease in wellbeing IG after 8 weeks; Sig. increase in sleep duration at 4 weeks (p=.033) and not Sig. increase at 8 weeks; Sig. improvement in sleep quality supported by the improvement wellbeing (p=.004) |
| Charron M et al., 2025 [26] | Sig. decrease in values of RR (p=.002) and anxiety score (p=.042) in MG; Sig. improvement in satisfaction scores in MG for patient (p=.0002) and the physician (p<.0001) |
| Hoegholt NF et al., 2024 [27] | Sig. difference in adherence to sleep hygiene between groups (p=.007) |
| Lee AL et al., 2024 [28] | No Sig. results were found in the study |
| Wakana K et al., 2022 [29] | No Sig. results were found in the study |
| Yang HF et al.,2024 [30] | Sig. in Post-test BAI-C score experimental group (p<.0001) |
| Harper FWK et al., 2023 [31] | Sig. improvement in positive mood and reduction of negative mood and distress (but not pain) from pre- to post-int. (p<.05); Selective benefit for some patients on the basis of relationship (p=.032) and employment (p=.029 |
| Aksu Ç , 2023 [32] | Sig. decrease of STAI-S and VAS-Pain scores IG vs the scores of CG (p<.05) after music intervention; Sig. increase in VAS Comfort scores IG with scores of CG (p<.05) after music intervention; Sig. increase in easiness of procedure IG after music intervention (p<.05) |
| Azi LMTA et al., 2021 [33] | Sig. reduction in anxiety in MG patients after surgery (p=.023)Less need for additional medication for intraoperative sedation in MG (p=.004) |
| Wang J et al., 2024 [34] | Sig. improvement of STAI in MIG vs CG (p<.001) |
| Sun DJ et al., 2022 [35] | Sig. decrease of anxiety after colonoscopy in piano group (p<.05); Sig. difference in satisfaction of colonoscopy process, pain management and overall service satisfaction than those of CG (p<.05); More pleasure in listening to music in next examination by patients (p<.001); |
| Jacquier S et al.,2022 [36] | No Sig. results were found in the study |
| Pampel J et al., 2024 [37] | Sig. reduction in NST at second timepoint IG vs CG (p<.01) |
| Başkurt H et al., 2024 [38] | Sig. difference between groups in terms of acceleration number (p<.005); Sig. increase in rate of reactive NST levels IG (p<.05); Sig. |

| | |
|---|---|
| | difference in STAI scale (higher CG) between groups at post-test (p<.01) |
| Öztürk FU et al., 2023 [39] | Sig. decrease in anxiety values in groups after biopsy (p<.05); Sig. reduction in state anxiety levels of MG (p=.024); Sig. decrease in pain perception (p<.05) and increase in patient satisfaction (p<.05) through the implementation of music |
| Chen YB et al., 2021 [40] | Sig. improvement in state anxiety IG vs CG (p=.01); Sig. increase of overall satisfaction IG vs the CG after six weeks postoperatively (p=.03) |
| Agus Y et al., 2025 [41] | Sig. decrease in ASSQ post-test scores than the pretest scores for groups (p<.05) |
| Fleckenstein FN et al., 2025 [42] | Sig. decrease in anxiety levels in patients in MG as vs patients CG (p<.001) |
| Petrovsky DV et al., 2023 [43] | Improvement sleep outcomes in groups after onset, small effect size for sleep duration post-intervention |
| Mir IA et al., 2021 [44] | Sig. reduction in SBP (p<.001) and HR (p=.002) in MG; Sig. difference in reduction in mean SBP (p<.001),HR (p=.006) between groups |
| Calamassi D et al., 2022 [45] | Decrease median values of STAI X1 in all groups from T0 to T1 (Group 1: p=.000; Group 2: p=.001 p=.028); Reduction mean values of respiratory rate and SBP at T1 in group 2 (p=.000; p=.031) |
| Lázaro-García A et al., 2024 [46] | Sig. in symptoms burden in alloSCT and in patient autoSCT in ML groups after ML sessions (p=.002 and p<.001,); Sig.diff. in proportion of satisfactory sessions for MLG (p<.001); Sig. diff. in satisfaction in MLG (p<.001) |
| Coşkun Ç et al., 2025 [47] | Sig. difference in median VAS score in MG and CG (p<.001); Sig. diff. in median s-STAI score in MG and CG (p<.001) |
| Hillebrand MC et al., 2023 [48] | Sig. decrease in BPSD during Individualized Music Listening (e.g. IML) (p=.040); Sig. interindividual variation in pre-session slope (p<.01) and post-session slope (p<.05); Sig. negative covariance between the intercept and the pre-session slope (p<.05); Sig. interindividual variation in intercept (p<.001); |
| Hakim A et al., 2025 [49] | Sig. reduction of anxiety score and the number of breaths per minute of hospitalized children (p ≤.01); Sig. decrease in vital signs except body temperature in test group (p ≤.01) |

| | |
|---|---|
| Durgun H et al., 2021 [50] | Sig. difference between groups, due to the decrease pain scores in MG (p<.001) |
| Schaal NK et al.,2021 [51] | Sig. reduction of SBP (p=.002) and HR (p=.035) from beginning of surgery to skin suture in MG, whereas the CG did not |
| Miladi S et al.,2024 [52] | Sig. reduction of HR in MG (p=.02); Sig. decrease of STAI in MG (p=.018) |
| Brix LD et al., 2022 [53] | No Sig. results were found in the study |
| Bürlukkara S et al., 2024 [54] | Sig. decrease in HR in MG (p=.02); Sig. decrease in VAS score in MG (p=.021); Sig. difference in STAI score between groups (p<.001) |
| Kaur H et al., 2022 [55] | Sig. decrease of HR in MG at 10 minutes of assessment (p=.003) and highly Sig. (p<.001) for rest of time period; Sig. decrease Respiratory Rate (e.g. RR) in MG (p=.05) |
| Ferraz MCL et al., 2021 [56] | Sig. decrease in pain scores in MG (p<.001) |
| Baykan S et al., 2025 [57] | Sig. reduction in severity of pain IG vs the CG (p<.001); Sig. decrease in pain severity score IG (p<.001); Sig. difference (p<.001) in group-time interaction; Sig. decrease of distress level in the IG in second and third session (p<.001); |
| Ugurlu M et al., 2024 [58] | Sig. difference in Post-test BDI and PSQI scores between the IG and CG (p<.05); Sig. difference in post-test MRS total, somatic, psychological symptoms scores between the IG and CG (p<.05) |
| Tola YO et al., 2025 [59] | Sig. difference between groups in change in level of pain intensity at 24 hours (p<.001) and 48 hours (p=.007); Sig. difference between groups in change in level of preop anxiety (p=.005); Sig. difference between groups in change in level of satisfaction with care on the day of surgery (p=.009); Sig. difference between groups only in SBP (p=.034) and DBP (p=.036) readings at 48 hours after surgery |
| Inoue M et al., 2024 [60] | Sig. increase of total PHQ-9 scores IG than the CG (p<.01) |
| Baltacı N et al., 2023 [61] | Sig. decrease in prenatal distress levels IG than CG (p<.01); Sig decrease in prenatal distress levels in MG than in Listening Group (e.g. LG) (p<.05); Sig. increase in antenatal attachment levels IGs than CG (p<.01); Sig. increase in antenatal attachment levels in LG than in MG (p<.05) |
| Oyur Celik G et al., 2022 [62] | Sig. difference between the means of anxiety (p=.000) and pain (p=.001) of patients in experimental groups and CG after procedure; |

| | |
|---|---|
| | Sig. difference before and after procedure for DBP (p=.002) Band pulse wave velocity (p=.002) in vital signs between groups |
| Barcos- Munoz F et al., 2025 [63] | Sig. difference in SD1 and SD2 variables in MG during baseline period recording (p<.05) |
| Hillebrand MC et al., 2025 [64] | Sig. increase in goal attainment in the IG vs the CG (p=.004) |
| Sökmen Y et al., 2024 [65] | Sig. increase in basal fetal HR and count of fetal movements, the presence of acceleration, the NST results of pregnant women in MG than those of CG (p<.05); Sig. difference between the groups in terms of mean satisfaction scores (p<.05) |
| Esteban Pellicer LÁ et al., 2023 [66] | Sig. difference in degree of anxiety before and after surgery in baroque MG (p=.027) and classical MG (p=.044) |
| Cavnar Helvaci B et al., 2024 [67] | Significant decrease in pain scores (p=.000) |
| Çuhacı AB etal., 2024 [68] | Sig. decrease in PIPP-R scores in experimental groups during and after procedure than the PIPP-R scores of infants CG (p<.001); Sig. decrease in post-procedure and pre procedure PIPP-R scores (p<.05); Sig. difference in post-procedure PICS values of preterm infants experimental group to be lower than those of CG (p<.001); Sig. difference when the music applied to preterm infants during ROP examination resulted in a mean decrease 1.286 in PICS scores after procedure and before procedure (p<.05) |
| Esfahanian F et al., 2022 [69] | Sig. effect of music in preventing delirium (p=.016) |
| Toker E et al., 2021 [70] | Sig. reduction in anxiety scores and pain levels IGs in comparison to the CG (p<.001); Sig. decrease in pain levels on the second day IG 2 than those of IG 1 (p<.05); Sig. decrease in pain levels in all groups (p<.001); Sig. increase in anxiety scores CG on the second day (p<.05) and decrease in IG 2 (p<.001) |
| Aker N et al., 2024 [71] | Sig. difference in STAI-S IG (p=.006),vs no Sig. difference in CG; Sig. difference in the IG (p=.011) vs no Sig. difference in the CG in pre-test and post-test surgery specific anxiety scale |
| Gürkan O et al., 2024 [72] | Sig. difference in state anxiety scale, which represents the decrease in pre-procedure and post-procedure anxiety levels (SAS1-SAS2),among the groups (p=.01); Sig. difference between the two groups in terms of VAS score distribution (p=.01) |

| | |
|---|---|
| Huang YL et al., 2021 [73] | Sig. decrease at T2,T3,T4,of MAP and HR of MT group than the CG (p<.05); Sig. decrease in mYPAS-SF scores of Music Therapy (e.g. MT) group than the CG at T2 and T3 (p<.01); Sig. decrease in ICC score of MT group than the CG (p<.01) |
| Zhang Y et al., 2025 [74] | Sig. reduction in STAI-S scores in MG than CG (p<.001); Sig. increase in ACTH levels (p=.03) and cortisol levels (p=.003) CG than the MG |
| Torlak MS et al., 2025 [75] | Sig. difference in the Beck Anxiety Inventory, visual analog scale, Neck Disability Index, and SF-36 physical scores in intragroup comparisons in both groups (p<.05). Decrease in visual analog scale score of the participants in the MG compared to CG (p<.01) |
| Khan BA et al., 2025 [76] | No Sig. results were found in the study |
| Doğan P et al., 2025 [77] | Sig. difference in pain level after procedure in MGs (p<.001) |
| Pradit L et al., 2022 [78] | No Sig. results were found in the study |
| Hillebrand MC et al., 2024 [79] | No Sig. results were found in the study |
| Ezepue CO et al., 2023 [80] | Sig. decrease in anxiety scores in MG (p=.001) |

**Table S1. Detailed examination of the selected studies' results**
